# The revised cardiac risk index and 90-day mortality after non-cardiac surgery: a retrospective cohort study

**DOI:** 10.3389/fcvm.2026.1773496

**Published:** 2026-07-15

**Authors:** Guangqin Ren, Qing Xie, Xue Guo, Guanglin Sang, Danqing Ren, Zhiwei Li, Xiangmei Tan, Huibing Chen, Yang Song, Lijuan Dong

**Affiliations:** 1 The Tenth Clinical Medical College of Guangzhou University of Traditional Chinese Medicine, Zhongshan, China; 2 Zhongshan Hospital of Traditional Chinese Medicine, Zhongshan, China; 3 Information Engineering University, Zhengzhou, China; 4 Guangzhou University of Chinese Medicine, Guangzhou, China

**Keywords:** 90-day mortality, cohort study, non-cardiac surgery, postoperative outcomes, revised cardiac risk index, risk stratification

## Abstract

**Background:**

The Revised Cardiac Risk Index (RCRI) is widely used for predicting major cardiac complications after non-cardiac surgery, but its association with 90-day all-cause mortality is less well established. This study aimed to evaluate the independent association between RCRI scores and 90-day all-cause mortality in a large, diverse cohort of patients undergoing non-cardiac surgery.

**Methods:**

We conducted a retrospective cohort study using data from 54,933 adult patients who underwent non-cardiac surgery at a tertiary care center in Singapore between 2012 and 2016. RCRI scores were calculated based on six clinical variables and categorized into four classes. The primary outcome was 90-day all-cause mortality. Survival analysis was performed using Kaplan–Meier curves and multivariable Cox proportional hazards models, with adjustments for demographic and clinical covariates.

**Results:**

A total of 735 patients (1.3%) died within 90 days postoperatively. Kaplan–Meier analysis revealed significantly poorer survival in higher RCRI classes (log-rank *p* < 0.001). In the fully adjusted model, compared to RCRI Class I, the hazard ratios for 90-day mortality were 1.97 (95% CI: 1.54–2.52) for Class II, 1.93 (95% CI: 1.45–2.58) for Class III, and 3.08 (95% CI: 2.29–4.15) for Class IV (*p* for trend <0.001). Subgroup analyses confirmed consistent associations across age, sex, ASA class, and surgical priority groups.

**Conclusion:**

Higher RCRI scores are independently associated with increased 90-day mortality after non-cardiac surgery, demonstrating a clear dose–response relationship. These findings support the use of RCRI as a practical and effective tool for preoperative risk stratification in diverse surgical populations.

## Graphical Abstract

**Figure:**
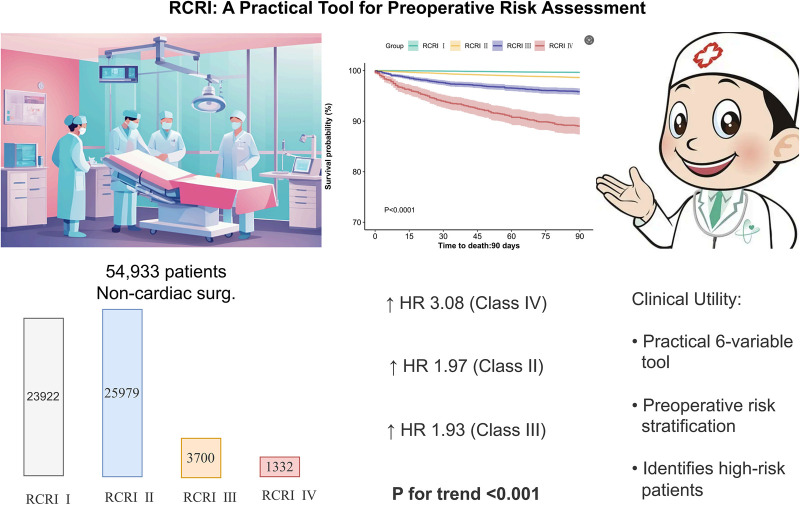

## 1 Introduction

Postoperative mortality serves as an important indicator for assessing the quality of surgical care (1). Although 30-day mortality has long been regarded as the standard endpoint in surgical outcomes research (2), growing evidence suggests that 90-day mortality provides a more comprehensive evaluation of surgical risk (3). This extended observation window is particularly important for patients with multiple comorbidities, whose physiological reserve may remain compromised beyond the early postoperative period (4).

In this context, practical and accurate preoperative risk stratification tools are crucial for identifying high-risk patients. The Revised Cardiac Risk Index (RCRI) is one of the most widely used risk stratification tools in clinical practice. Originally developed by Lee et al., the index was designed to assess the risk of major cardiac complications within 30 days following non-cardiac surgery (5). RCRI includes six easily obtainable clinical variables, making it particularly suitable for rapid clinical assessment (6). Beyond its original purpose of predicting cardiac-specific outcomes, subsequent studies have demonstrated a significant association between RCRI scores and all-cause mortality (1), suggesting its ability to reflect broader features of physiological vulnerability (7).

Evidence supporting the utility of RCRI has been confirmed across various surgical specialties. Previous studies have established a significant association between RCRI scores and short-term mortality in patients undergoing hip fracture surgery (8). Similarly, studies in patients undergoing elective colorectal cancer surgery have demonstrated a graded relationship between RCRI scores and 90-day mortality (9). These consistent findings across different surgical populations highlight the potential value of RCRI as a risk assessment tool in mixed surgical cohorts. These findings align with a broader research effort to predict postoperative death using diverse approaches, including machine learning models for emergency surgery (10), novel risk scores (11), and biomarkers such as high-sensitivity troponin (12). Moreover, studies consistently highlight the elevated mortality risk associated with specific high-risk states, such as perioperative COVID-19 infection (13) and liver cirrhosis (14), underscoring the need for tools that capture underlying physiological vulnerability. However, evidence remains limited regarding the association between RCRI and short-term postoperative mortality in general surgical (non-cardiac) patients, particularly in terms of short-term prognosis reflecting 90-day mortality risk (1).

Based on the existing literature, this study aims to investigate the association between RCRI scores and 90-day all-cause mortality in a large, diverse cohort of patients undergoing non-cardiac surgery. We hypothesize that higher RCRI scores are independently associated with an increased risk of 90-day mortality.

## 2 Methods

### 2.1 Data collection

This study constitutes a secondary analysis of a previously published retrospective cohort by Yilin Eileen Sim et al., with the original dataset publicly accessible through the Dryad Digital Repository (DOI: 10.1371/journal.pone.0182543). The cohort included patients who underwent non-cardiac surgical procedures at a tertiary care institution in Singapore from January 1, 2012, to October 31, 2016. Clinical data were extracted from the Sunrise Clinical Manager system and maintained in the Electronic Health Intelligence System (7), while mortality outcomes were linked with national electronic health records to ensure comprehensive follow-up. The original retrospective study protocol received ethical approval from the SingHealth Centralised Institutional Review Board (Reference: SingHealth CRB 2014/651/D). All patients were followed for 90 days postoperatively through linkage with national electronic health records. No loss to follow-up was observed for the primary outcome.

### 2.2 Subjects

**Figure 1:**
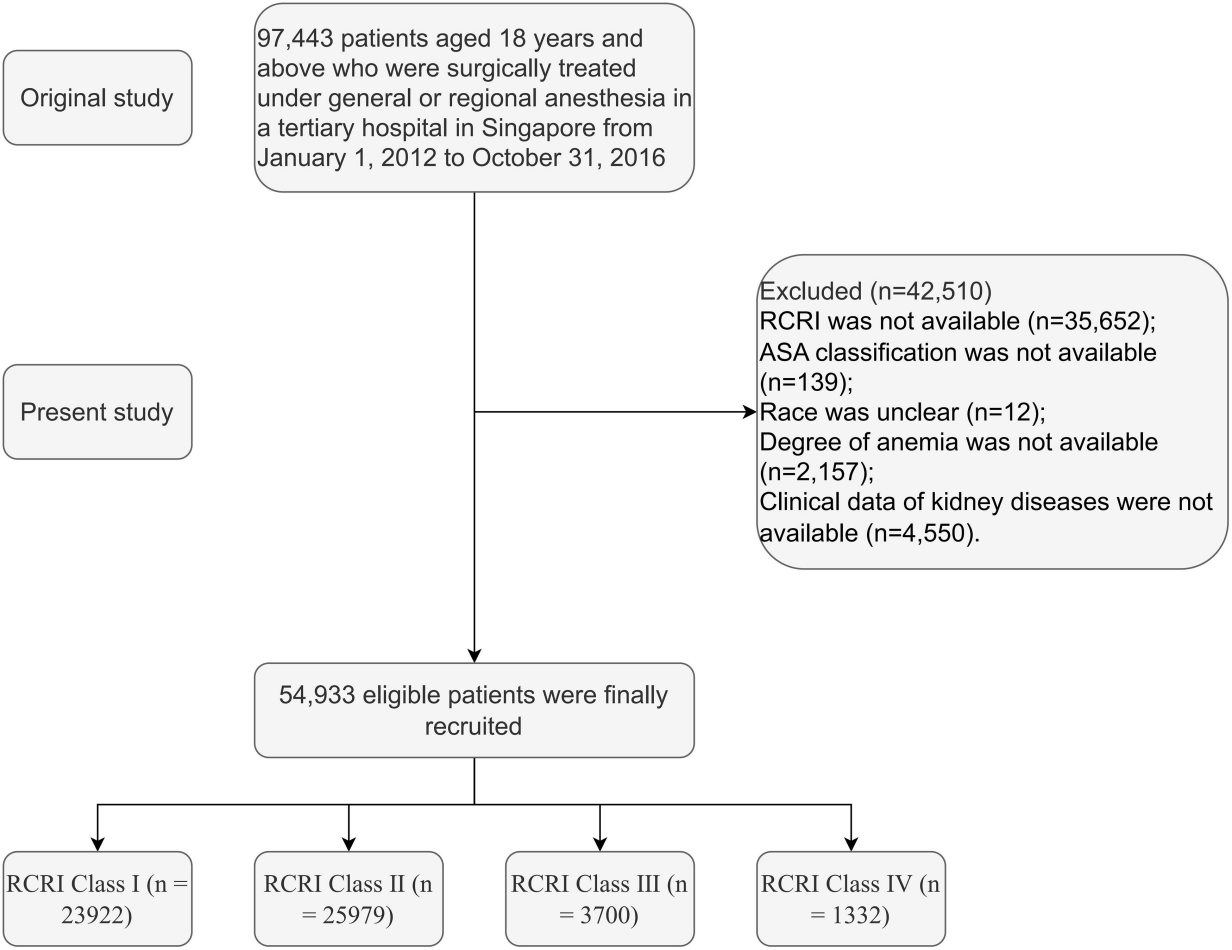
Patient selection flowchart.

The study initially screened 97,443 patients aged 18 years and above who underwent surgical procedures under general or regional anesthesia. After applying exclusion criteria for missing data on key study variables including RCRI components (*n* = 35,652) and important clinical covariates [ASA classification (*n* = 139), race (*n* = 12), degree of anemia (*n* = 2,157), and kidney disease status (*n* = 4,550)], the final cohort comprised 54,933 eligible patients (Figure 1).

Flow diagram illustrating the identification and inclusion of study participants. The initial cohort consisted of 97,443 adults undergoing non-cardiac surgery between 2012 and 2016. After excluding 42,510 patients due to missing data on key variables (RCRI components, ASA classification, race, anemia degree, and kidney disease status), the final analytical cohort comprised 54,933 patients.

### 2.3 Calculation of RCRI

The Revised Cardiac Risk Index (RCRI) was calculated based on six clinical variables: history of ischemic heart disease, history of congestive heart failure, history of cerebrovascular disease, diabetes mellitus requiring insulin therapy, preoperative serum creatinine >2 mg/dL, and high-risk surgery. One point was assigned for each variable present. According to the 2014 ESC/ESA Guidelines on non-cardiac surgery (15, 16), patients were stratified into four risk categories: Class I (0 points), Class II (1 point), Class III (2 points), and Class IV (≥3 points).

### 2.4 Statistical analysis

Categorical variables are presented as frequencies and percentages, and compared using the chi-square test. Survival analysis was conducted using the Kaplan–Meier method, with the log-rank test employed to compare survival distributions across groups. Univariate and multivariate Cox proportional hazards models were utilized to identify risk factors associated with 90-day mortality. Building on previous literature and clinical relevance, we constructed three sequential multivariable models: Model I (unadjusted), Model II (adjusted for demographic characteristics including age, sex, and race), and Model III [further adjusted for clinical covariates including ASA (American Society of Anesthesiologists) physical status, anesthesia type, surgical priority, and severity of preoperative anemia]. Categorization of variables such as age groups and anemia severity was based on commonly used clinical thresholds and prior literature to ensure clinical relevance and comparability. To evaluate the robustness of our findings, we performed subgroup analyses by stratifying patients according to age (<70 vs. ≥70 years), sex (male vs. female), ASA classification (1–2 vs. 3–6), surgical risk category (low, medium, high), anesthesia type, and surgical priority. Interaction effects were tested using multiplicative interaction terms in the Cox models. In addition to the primary subgroup analysis comparing RCRI Class IV with Classes I–III, supplementary subgroup analyses were conducted using alternative categorizations, including low-risk vs. high-risk groups (Classes I–II vs. Classes III–IV), and (2) lowest risk vs. all higher-risk groups (Class I vs. Classes II-III-IV). To assess the robustness and clinical interpretability of the findings. Given the presence of missing data in key variables, a complete-case analysis approach was adopted. Patients with missing information on RCRI components or selected covariates were excluded from the final analytical cohort. No multiple imputation procedures were performed. Alternative approaches such as multiple imputation were considered but not pursued, as the proportion of missing data exceeded 40% and the pattern of missingness could not be assumed to be missing at random. The baseline characteristics of excluded vs. included patients could not be directly compared because the excluded patients lacked key variables required for RCRI calculation. All statistical analyses were performed using R software (version 3.3.2; R Foundation for Statistical Computing, Vienna, Austria) and Free Statistics software (version 2.3.0). A two-sided *p*-value <0.05 was considered statistically significant.

## 3 Results

### 3.1 Baseline characteristics

The final cohort comprised 54,933 patients, all of whom showed statistically significant differences in baseline characteristics across RCRI classes (all *p* < 0.001). Patients with higher RCRI scores were more likely to be male, older, and to carry a greater burden of comorbidities. The proportion of patients with an ASA physical status classification of III or higher increased markedly from 7.0% in RCRI Class I to 90.1% in Class IV. Similarly, the prevalence of stage 4 kidney disease demonstrated a substantial gradient, rising from 0.4% to 49.5% across the RCRI strata (Table 1).

**Table 1:** Baseline characteristics of the study population stratified by revised cardiac risk Index (RCRI) class.

Variables	Total (*n* = 54,933)	RCRI class I (*n* = 23,922)	RCRI class II (*n* = 25,979)	RCRI class III (*n* = 3,700)	RCRI class IV (*n* = 1,332)	*P* value
Male, *n* (%)	26,933 (49.0)	13,290 (55.6)	10,754 (41.4)	2,090 (56.5)	799 (60.0)	<0.001
Age, *n* (%)						<0.001
18–29 yrs	5,034 (9.2)	3,092 (12.9)	1,918 (7.4)	20 (0.5)	4 (0.3)	
30–49 yrs	14,938 (27.2)	8,116 (33.9)	6,439 (24.8)	306 (8.3)	77 (5.8)	
50–69 yrs	24,604 (44.8)	10,020 (41.9)	12,131 (46.7)	1,781 (48.1)	672 (50.5)	
≥70 yrs	10,357 (18.9)	2,694 (11.3)	5,491 (21.1)	1,593 (43.1)	579 (43.5)	
Race, *n* (%)						<0.001
Chinese	40,071 (72.9)	16,813 (70.3)	19,546 (75.2)	2,772 (74.9)	940 (70.6)	
Malay	5,224 (9.5)	2,363 (9.9)	2,304 (8.9)	373 (10.1)	184 (13.8)	
Indian	4,672 (8.5)	2,407 (10.1)	1,797 (6.9)	327 (8.8)	141 (10.6)	
Others	4,966 (9.0)	2,339 (9.8)	2,332 (9.0)	228 (6.2)	67 (5.0)	
ASA physical status, *n* (%)						<0.001
Class Ⅰ	12,406 (22.6)	7,593 (31.7)	4,803 (18.5)	9 (0.2)	1 (0.1)	
Class Ⅱ	32,508 (59.2)	14,654 (61.3)	16,496 (63.5)	1,227 (33.2)	131 (9.8)	
Class Ⅲ	9,234 (16.8)	1,609 (6.7)	4,333 (16.7)	2,274 (61.5)	1,018 (76.4)	
Class Ⅳ	769 (1.4)	63 (0.3)	341 (1.3)	184 (5.0)	181 (13.6)	
Class Ⅴ	16 (0.0)	3 (0.0)	6 (0.0)	6 (0.2)	1 (0.1)	
Type of anesthesia, *n* (%)						<0.001
GA	46,839 (85.3)	21,678 (90.6)	21,523 (82.8)	2,760 (74.6)	878 (65.9)	
RA	8,094 (14.7)	2,244 (9.4)	4,456 (17.2)	940 (25.4)	454 (34.1)	
Priority of Surgery, *n* (%)						<0.001
Elective	44,732 (81.4)	19,507 (81.5)	21,521 (82.8)	2,816 (76.1)	888 (66.7)	
Emergency	10,201 (18.6)	4,415 (18.5)	4,458 (17.2)	884 (23.9)	444 (33.3)	
Surgical risk, *n* (%)						<0.001
Low-risk	27,214 (49.5)	23,922 (100.0)	2,401 (9.2)	631 (17.1)	260 (19.5)	
Medium-risk	24,811 (45.2)	0 (0.0)	21,352 (82.2)	2,591 (70.0)	868 (65.2)	
High-risk	2,908 (5.3)	0 (0.0)	2,226 (8.6)	478 (12.9)	204 (15.3)	
Degree of preoperative anemia, *n* (%)						<0.001
None	39,897 (72.6)	19,485 (81.5)	18,309 (70.5)	1,730 (46.8)	373 (28.0)	
Mild	8,155 (14.8)	2,754 (11.5)	4,117 (15.8)	916 (24.8)	368 (27.6)	
Moderate	6,639 (12.1)	1,616 (6.8)	3,443 (13.3)	1,020 (27.6)	560 (42.0)	
Severe	242 (0.4)	67 (0.3)	110 (0.4)	34 (0.9)	31 (2.3)	
Grade of kidney disease, *n* (%)						<0.001
Stage 1	33,087 (60.2)	15,771 (65.9)	16,002 (61.6)	1,125 (30.4)	189 (14.2)	
Stage 2	16,203 (29.5)	7,037 (29.4)	7,734 (29.8)	1,194 (32.3)	238 (17.9)	
Stage 3	3,462 (6.3)	1,011 (4.2)	1,632 (6.3)	574 (15.5)	245 (18.4)	
Stage 4–5	2,181 (4.0)	103 (0.4)	611 (2.4)	807 (21.8)	660 (49.5)	
CVA, *n* (%)	1,531 (2.8)	0 (0.0)	329 (1.3)	724 (19.6)	478 (35.9)	<0.001
IHD, *n* (%)	4,066 (7.4)	0 (0.0)	1,048 (4.0)	1,933 (52.2)	1,085 (81.5)	<0.001
CHF, *n* (%)	752 (1.4)	0 (0.0)	62 (0.2)	233 (6.3)	457 (34.3)	<0.001
Insulin use for diabetes mellitus, *n* (%)	1,827 (3.3)	0 (0.0)	504 (1.9)	697 (18.8)	626 (47.0)	<0.001
Cr > 2 mg/dL, *n* (%)	1,870 (3.4)	0 (0.0)	458 (1.8)	744 (20.1)	668 (50.2)	<0.001
Died within 90 day postoperatively, *n* (%)	735 (1.3)	82 (0.3)	353 (1.4)	154 (4.2)	146 (11.0)	<0.001

Data are presented as *n* (%). Categorical variables were compared using the chi-square test.

RCRI, Revised Cardiac Risk Index; ASA, American Society of Anesthesiologists; GA, general anesthesia; RA, regional anesthesia; CVA, cerebrovascular accident; IHD, ischemic heart disease; CHF, congestive heart failure; Cr, creatinine. *P* values indicate overall differences across the four RCRI classes.

### 3.2 Survival analysis

**Figure 2:**
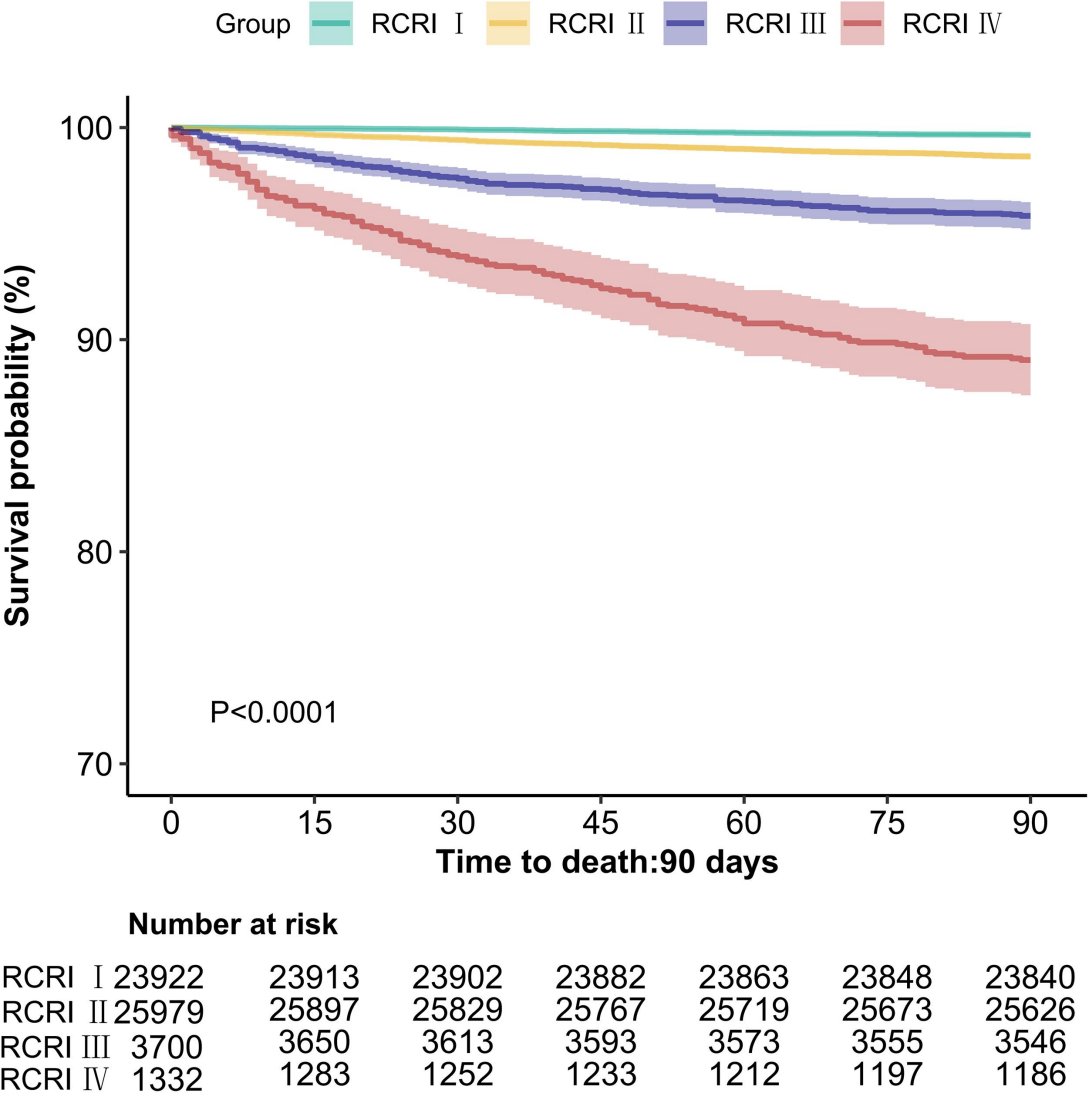
Kaplan–Meier survival curves for 90-Day mortality, stratified by revised cardiac risk Index (RCRI) class.

Kaplan–Meier analysis revealed significantly different survival patterns among RCRI classes (log-rank *p* < 0.001). Patients in RCRI Class I maintained the highest survival probability throughout the 90-day follow-up period, while those in Class IV demonstrated the most rapid decline in survival, particularly during the initial postoperative period (Figure 2).

Time-to-event analysis shows unadjusted survival probabilities over the 90-day postoperative period. The four curves represent RCRI Class I (T1) through Class IV (T4). A log-rank test confirmed statistically significant separation of the survival distributions (*p* < 0.001). The number of patients at risk at each 15-day interval is shown in the table below the figure.

### 3.3 Risk factors for 90-day postoperative mortality

#### 3.3.1 Univariable analysis

In univariable Cox regression, RCRI showed a strong graded association with 90-day mortality. Compared to RCRI Class I, the hazard ratios were 3.98 (95% CI 3.13–5.07) for Class II, 12.42 (95% CI 9.50–16.23) for Class III, and 34.02 (95% CI 25.96–44.59) for Class IV (all *p* < 0.001). Other significant predictors included higher ASA classification, emergency surgery, elevated surgical risk, anemia severity, and individual RCRI components (Table 2).

**Table 2:** Univariable Cox regression analysis of factors associated with 90-Day mortality.

Item	HR (95%CI)	*P* (Wald's test)
RCRI		
Class I	Reference	
Class II	3.98 (3.13,5.07)	<0.001
Class III	12.42 (9.5,16.23)	<0.001
Class IV	34.02 (25.96,44.59)	<0.001
Gender		
Female	Reference	
Male	1.39 (1.2,1.61)	<0.001
Age		
18–29 yrs	Reference	
30–49 yrs	1.63 (0.88,3.03)	0.123
50–69 yrs	5.45 (3.06,9.7)	<0.001
≥70 yrs	14.28 (8.03,25.39)	<0.001
Race		
Chinese	Reference	
Malay	1.15 (0.91,1.44)	0.237
Indian	0.92 (0.7,1.19)	0.515
Others	0.41 (0.29,0.6)	<0.001
ASA physical status		
Class I	Reference	
Class II	5.18 (2.81,9.55)	<0.001
Class III	51.15 (28.1,93.09)	<0.001
Class IV	272.68 (148.02,502.32)	<0.001
Class V	449.84 (156.29,1,294.72)	<0.001
Type of anesthesia		
GA	Reference	
RA	1.36 (1.13,1.64)	<0.001
Priority of Surgery		
Elective	Reference	
Emergency	4.12 (3.57,4.76)	<0.001
Surgical risk		
Low-risk	Reference	
Medium-risk	2.08 (1.76,2.46)	<0.001
High-risk	6.21 (5,7.71)	<0.001
Degree of preoperative anemia		
None	Reference	
Mild	3.38 (2.74,4.18)	<0.001
Moderate	10.07 (8.48,11.95)	<0.001
Severe	28.91 (20.13,41.53)	<0.001
Grade of kidney disease		
Stage 1	Reference	
Stage 2	1.06 (0.87,1.29)	0.582
Stage 3	4.12 (3.33,5.11)	<0.001
Stage 4–5	10.83 (9.01,13.02)	<0.001
CVA		
No	Reference	
Yes	3.75 (2.93,4.79)	<0.001
IHD		
No	Reference	
Yes	5.97 (5.11,6.97)	<0.001
CHF		
No	Reference	
Yes	9.14 (7.23,11.54)	<0.001
Insulin use for diabetes mellitus		
No	Reference	
Yes	3.92 (3.13,4.91)	<0.001
Cr > 2 mg/dL		
No	Reference	
Yes	8.94 (7.54,10.62)	<0.001

HR, hazard ratio; CI, confidence interval; RCRI, Revised Cardiac Risk Index; ASA, American Society of Anesthesiologists; GA, general anesthesia; RA, regional anesthesia; CVA, cerebrovascular accidents; IHD, ischemic heart disease; CHF, congestive heart failure; Cr, creatinine.

#### 3.3.2 Multivariable analysis

After sequential adjustment for potential confounders, RCRI remained independently associated with 90-day mortality. In the fully adjusted model (Model III), the hazard ratios were 1.97 (95% CI 1.54–2.52) for Class II, 1.93 (95% CI 1.45–2.58) for Class III, and 3.08 (95% CI 2.29–4.15) for Class IV compared to Class I. A significant dose-response relationship persisted (*p* for trend <0.001), confirming the independent predictive value of RCRI beyond traditional risk factors (Table 3).

**Table 3:** Multivariable Cox regression analysis of the association between RCRI and 90-Day mortality.

RCRI class	Model I		Model II		Model III	
	HR (95% CI)	*P*-value	HR (95% CI)	*P*-value	HR (95% CI)	*P*-value
RCRI Class I	1(Ref)		1(Ref)		1(Ref)	
RCRI Class II	3.98 (3.13∼5.07)	<0.001	3.44 (2.7∼4.39)	<0.001	1.97 (1.54∼2.52)	<0.001
RCRI Class III	12.42 (9.5∼16.23)	<0.001	7.47 (5.67∼9.83)	<0.001	1.93 (1.45∼2.58)	<0.001
RCRI Class IV	34.02 (25.96∼44.59)	<0.001	19.54 (14.79∼25.83)	<0.001	3.08 (2.29∼4.15)	<0.001
*P* for trend	3.13 (2.91∼3.37)	<0.001	2.55 (2.35∼2.75)	<0.001	1.33 (1.22∼1.45)	<0.001

Model I: Unadjusted. Model II: Adjusted for age, sex, and race. Model III: Additionally adjusted for ASA physical status, anesthesia type, priority of surgery, and degree of preoperative anemia. The *P* for trend was calculated by treating the RCRI classes as an ordinal variable in the regression models.

HR, hazard ratio; CI, confidence interval.

### 3.4 Subgroup analysis

**Figure 3:**
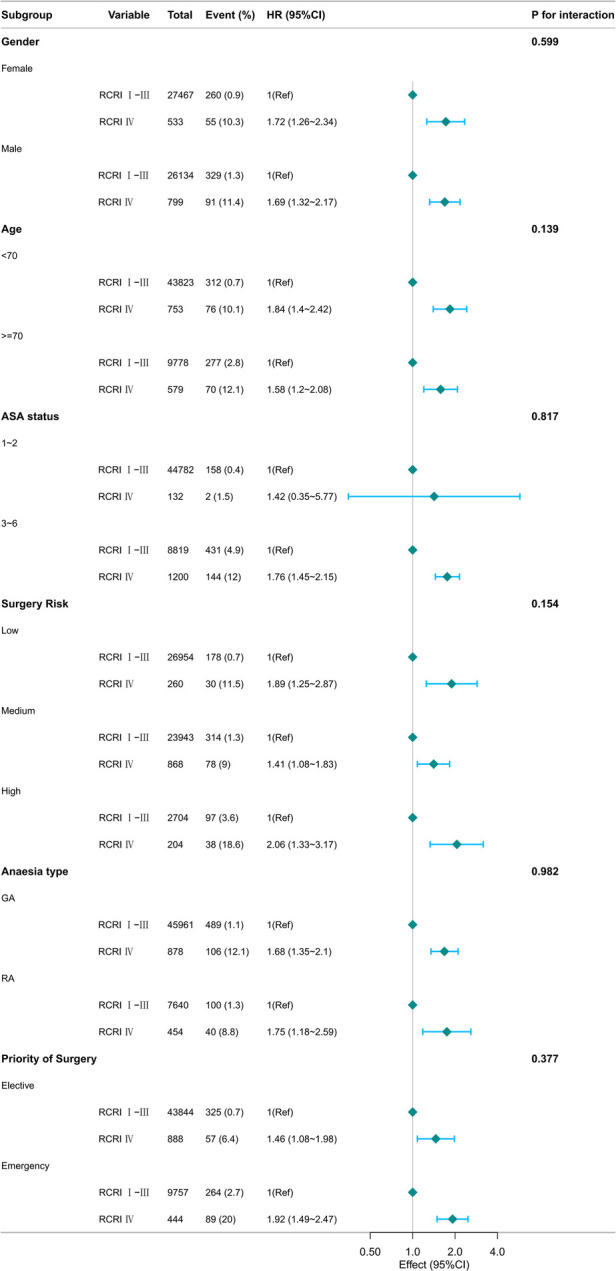
Forest plot for subgroup analysis of the association between RCRI class IV and 90-Day mortality.

Subgroup analyses demonstrated consistent associations between RCRI Class IV and increased mortality risk across all patient strata, including different genders, age groups, ASA status categories, surgical risk levels, anesthesia types, and surgery priorities (Figure 3). Formal interaction tests revealed no significant effect modification by any subgroup variable (all *p* for interaction > 0.05), supporting the robustness and generalizability of the primary findings.

To further enhance clinical interpretability, we conducted two additional sets of subgroup analyses using alternative groupings. First, comparing low-risk (Classes I-II) vs. high-risk (Classes III-IV) patients, the fully adjusted model demonstrated a hazard ratio of 1.34 (95% CI: 1.14–1.57, *p* < 0.001) for 90-day mortality. Second, comparing Class I (lowest risk) vs. Classes II-III-IV (all higher-risk patients), the hazard ratio was 2.06 (95% CI: 1.61–2.62, *p* < 0.001). In both supplementary analyses, subgroup stratification across age, sex, ASA classification, anesthesia type, and surgical priority showed consistent associations, with no significant effect modification (all *p* for interaction > 0.05). Notably, subgroup analysis by surgical risk category could not be performed for the Class I vs. II-III-IV comparison because all RCRI Class I patients were exclusively classified as low surgical risk (Table 1), leading to perfect collinearity.

These supplementary results are presented in Supplementary Tables S1, S2 and Supplementary Figures S1, S2.

The plot displays hazard ratios (HRs, solid squares) and 95% confidence intervals (horizontal lines) for the association of RCRI Class IV (vs. Classes I-III) with 90-day mortality across various patient subgroups. The size of the data markers corresponds to the precision of the estimate. Formal tests for interaction showed no significant effect modification by any subgroup variable (all *p* for interaction > 0.05), indicating a consistent association. GA, general anesthesia; RA, regional anesthesia.

## 4 Discussion

This large-scale cohort study demonstrates a significant, independent, and graded association between the Revised Cardiac Risk Index (RCRI) and 90-day all-cause mortality after non-cardiac surgery. After full adjustment for key confounders, patients in RCRI Class IV had a 3.08-fold higher mortality risk compared to those in Class I, with a consistent trend across subgroups. These findings confirm that the RCRI robustly captures a gradient of physiological vulnerability that translates into early postoperative mortality risk. Additional subgroup analyses using clinically intuitive categorizations yielded consistent results, further supporting the robustness of the observed associations.

The Revised Cardiac Risk Index (RCRI) demonstrates predictive capacity for post-noncardiac surgery mortality by effectively assessing patients' broad physiological vulnerability beyond cardiac-specific factors. The six integrated clinical variables—including heart failure, renal insufficiency, and insulin-dependent diabetes—collectively reflect chronic multi-organ functional reserve decline and may partially reflect underlying systemic inflammatory status. This is corroborated by large-scale studies showing that perioperative acute kidney injury (17) and myocardial injury (18) are among the strongest drivers of postoperative death. Furthermore, the systemic inflammatory state implied by RCRI components aligns with findings that elevated postoperative inflammatory markers [e.g., neutrophil-to-lymphocyte ratio (19), C-reactive protein (20)] are independently associated with in-hospital mortality. When surgically stressed, such patients exhibit significantly compromised physiological homeostasis, increasing susceptibility not only to cardiac events but also to infectious complications and respiratory failure (17, 21), thereby elevating mortality risk.

This mechanism aligns with findings from Sim et al., where systemic indicators like anemia and high RDW strongly correlated with mortality (7), jointly emphasizing the prognostic significance of preoperative physiological fragility. Furthermore, intraoperative technical factors and institutional resources may also influence postoperative mortality, particularly in complex or emergency settings. While the absence of such variables in the present study represents a limitation, it also underscores the role of RCRI as a purely preoperative risk stratification tool, which may enhance its applicability in clinical decision-making. Whereas traditional tools like the 18-variable POSSUM score (22), 17-variable Charlson Comorbidity Index (23–25) face clinical limitations due to complexity or subjectivity, RCRI offers a practical alternative: it requires only six objectively measurable preoperative variables, balancing accurate physiological vulnerability assessment with clinical feasibility for efficient risk stratification.

Developed by Lee et al., the Revised Cardiac Risk Index (RCRI) is used to assess the risk of 30-day cardiac events and mortality following non-cardiac surgery (5). Its predictive value has been confirmed by numerous studies. Lindenauer et al. found a proportional relationship between RCRI scores and in-hospital mortality, with higher scores associated with increased mortality (26). A meta-analysis by Ford et al. validated the earlier conclusion that RCRI can predict adverse cardiac events after non-cardiac surgery. However, except for a subgroup analysis in vascular surgery where RCRI showed lower predictive value, they did not distinguish between different types of non-cardiac surgery (27).

Recent studies continue to support the clinical utility of RCRI. Hao et al. confirmed a significant association between RCRI and 1-year mortality, which remained consistent regardless of gender, age, or anesthesia type (1). Hulme et al. demonstrated that RCRI is also applicable to hip fracture surgery patients, with high RCRI scores significantly associated with increased 30- and 90-day postoperative mortality (9). Similarly, Forssten et al. showed that high RCRI scores (≥2) were significantly correlated with 30- and 90-day mortality in patients undergoing hip fracture surgery (8). Matthay et al. reported that intraoperative transfusion was associated with a 2–4-fold increased risk of 30-day major adverse cardiovascular events in patients undergoing infrainguinal revascularization, and this association remained significant after adjusting for RCRI and other risk factors (9). Bass et al. found that elevated RCRI scores were independently associated with in-hospital mortality and complication risks in elderly patients with rib fractures, with scores of 2 and 3 increasing adjusted mortality risks by 1.72-fold and 2.45-fold, respectively (28). In a nationwide cohort of elective colon cancer surgery patients, Hulme et al. observed a linear increase in 90-day postoperative mortality with rising RCRI scores, where patients with RCRI 4 had a 4.2-fold higher mortality than those with RCRI 1 (9). Hu et al. reported that perioperative myocardial injury was significantly associated with 30-day and long-term mortality in elderly Chinese orthopedic surgery patients, with RCRI scores ≥2 increasing the risk of adverse outcomes by 5.05-fold (29). Bronheim et al. demonstrated that RCRI predicts postoperative myocardial infarction and cardiac arrest in patients undergoing posterior lumbar decompression, with its discriminative ability surpassing that of ASA classification (30). Hao et al., in a single-center retrospective study, found that patients with RCRI Class IV had more than twice the 1-year postoperative mortality risk compared to Class I patients (adjusted HR 2.14), with the association remaining consistent across gender, age, and anesthesia subgroups (1). Palamuthusingam et al. observed that RCRI scores were significantly associated with 30-day major adverse cardiovascular events in chronic kidney replacement therapy patients undergoing elective general surgery, supporting its use for perioperative cardiac risk assessment in this high-risk population (31). Vetrugno et al., in a multicenter study of elderly hip fracture patients, noted that traditional cardiovascular scoring systems, including RCRI, may underestimate postoperative complication risks, suggesting the need for combined assessment approaches in special populations for more comprehensive preoperative risk stratification (32). The cumulative evidence thus affirms the utility of RCRI in various surgical contexts. Our study contributes to this body of knowledge by specifically validating its association with 90-day all-cause mortality in a broad, general non-cardiac surgery cohort. This focus addresses a notable gap, as much of the contemporary literature on postoperative mortality prediction has centered on either developing novel machine learning models or complex risk scores (10, 11), or on examining specific high-risk subgroups such as trauma patients (33) or those with cirrhosis (14).

This study focuses on 90-day mortality as the primary endpoint, and the results indicate that patients with high RCRI scores experience significantly elevated mortality risks early in the postoperative period. This finding not only validates the early predictive value of RCRI but also suggests that mortality risk is particularly concentrated within the first 90 days for high-risk patients.

Although we adjusted for multiple demographic and clinical covariates, including ASA classification, anemia severity, and surgical characteristics, residual confounding is likely to persist due to the observational nature of the study. Patients with higher RCRI scores were not only at increased cardiovascular risk but also tended to be older and have a greater burden of comorbidities, suggesting that RCRI may partially function as a surrogate marker of overall physiological vulnerability rather than an isolated cardiac risk measure. This is further supported by the attenuation of hazard ratios after multivariable adjustment and the relatively similar effect estimates observed for RCRI Class II and Class III in the fully adjusted model. These findings suggest that a substantial proportion of the crude risk gradient may be explained by coexisting comorbidities and other measured covariates. Therefore, the remaining association between RCRI and 90-day mortality should be interpreted with caution as a non-causal relationship, reflecting risk stratification rather than direct mechanistic effect. In addition, other potentially important factors, such as frailty, functional status, detailed severity of comorbidities, intraoperative variables, and socioeconomic factors, were not captured in the dataset, which may further contribute to residual confounding.

The strengths of this study lie in its use of real-world data from a large cohort of non-cardiac surgery patients and its substantial sample size. Additionally, the assessment tool, RCRI, relies on only six routinely available clinical variables, which facilitates rapid and practical preoperative risk stratification. Nevertheless, several limitations must be acknowledged. First, a substantial proportion of patients (approximately 43.6%) were excluded due to missing data on key variables, and a complete-case analysis approach was used. This may introduce selection bias if the excluded patients differ systematically from those included in the analysis. For instance, patients with missing data may represent more complex or emergent clinical scenarios or reflect differences in patient severity and care processes. As a result, the observed associations between RCRI and 90-day mortality may be subject to bias, potentially leading to either underestimation or overestimation of the true effect size. Moreover, the exclusion of a large number of patients may limit the generalizability of our findings. Future studies using multiple imputation or sensitivity analyses would be valuable to confirm the robustness of these findings. Second, the retrospective observational design is inherently susceptible to unmeasured confounding and limits causal inference. Third, reliance on data derived from hospital electronic records and national registries means that collected variables were primarily chronic disease indicators, with limited laboratory or detailed clinical data, which could introduce residual confounding. In particular, in patients with underlying malignancies, especially those with advanced or end-stage disease, postoperative mortality may be influenced by disease progression rather than perioperative risk alone, thereby acting as a potential source of residual confounding. Fourth, the study was conducted within a single tertiary center in Singapore, and its distinct demographic profile may affect the generalizability of our findings to other populations. Fifth, another limitation is the lack of detailed procedure-level classification in the dataset. Although surgical risk category and surgical priority were included as proxies for procedural complexity and urgency, more granular distinctions between surgical types (e.g., orthopedic, abdominal, vascular, or thoracic procedures) were not available. Given that the prognostic performance of RCRI may vary across different surgical contexts, future studies incorporating detailed procedural classifications are warranted to further assess its applicability across specific surgical populations. Finally, sensitivity analyses were not performed in this study, and future studies exploring alternative model specifications may help to further validate the robustness of these findings. Additionally, while RCRI effectively captures baseline vulnerability, it does not incorporate dynamic perioperative factors known to affect outcomes, such as intraoperative hemodynamics (21) or the occurrence of major bleeding (34), which future integrated models may consider. Therefore, external validation across diverse healthcare settings is warranted.

In conclusion, our study validates the RCRI as a practical tool for stratifying 90-day mortality risk prior to non-cardiac surgery. The consistent dose-response relationship supports its integration into routine preoperative assessment to identify high-risk patients who may benefit from intensified perioperative monitoring and management. To build upon this work, future research should focus on (1) developing integrated models that combine RCRI with dynamic biomarkers (e.g., hematological indices) to improve predictive precision; (2) designing and evaluating tailored perioperative care bundles for patients with RCRI ≥ 3; and (3) externally validating these findings in diverse healthcare settings to confirm generalizability. Such efforts will advance precision perioperative medicine and potentially improve outcomes for surgical patients.

## 5 Conclusion

This study confirms that the Revised Cardiac Risk Index (RCRI) is an independent predictor of 90-day mortality after non-cardiac surgery and shows a significant graded association. These results support its clinical application as a routine preoperative risk assessment tool. Future prospective studies are needed to validate these findings, develop independently associated models, and assess the efficacy of individualized management protocols in diverse, multicenter settings.

## Data Availability

Publicly available datasets were analyzed in this study. This data can be found here: https://doi.org/10.5061/dryad.5772v.

## Supplementary material

Supplementary Table S1 Multivariable Cox regression for RCRI I-II vs III-IV.

Supplementary Table S2 Multivariable Cox regression for RCRI I vs II-III-IV.

Supplementary Figure S1 Forest plot for RCRI I-II vs III-IV subgroup analyses.

Supplementary Figure S2 Forest plot for RCRI I vs II-III-IV subgroup analyses.
