# Supplementary material for: The revised cardiac risk index and 90-day mortality after non-cardiac surgery: a retrospective cohort study
Source: Front Cardiovasc Med. 2026 Jul 15;13:1773496. doi: 10.3389/fcvm.2026.1773496 (PMC13417123; doi:10.3389/fcvm.2026.1773496)
Supplement: Supplementary Table S1 — Multivariable Cox regression for RCRI I-II vs III-IV. [file Datasheet1.docx]

**Supplementary Table S1. Multivariable Cox Regression Analysis of the Association Between RCRI (Classes I-II vs. III-IV) and 90-Day Mortality**

| RCRI Class | Model Ⅰ | | Model Ⅱ | | Model Ⅲ | |
| --- | --- | --- | --- | --- | --- | --- |
|  | HR  (95% CI) | *P*-value | HR  (95% CI) | *P*-value | HR  (95% CI) | *P*-value |
| Classes I-II | 1(Ref) |  | 1(Ref) |  | 1(Ref) |  |
| Classes III-IV | 7.05 (6.08~8.16) | <0.001 | 4.4 (3.77~5.13) | <0.001 | 1.34 (1.14~1.57) | <0.001 |

Model I: Unadjusted. Model II: Adjusted for age, sex, and race. Model III: Additionally adjusted for ASA physical status, anesthesia type, priority of surgery, and degree of preoperative anemia. The P for trend was calculated by treating the RCRI classes as an ordinal variable in the regression models. HR, hazard ratio; CI, confidence interval.

**Supplementary Table S2.** **Multivariable Cox Regression Analysis of the Association Between RCRI and 90-Day Mortality**

| RCRI Class | Model Ⅰ | | Model Ⅱ | | Model Ⅲ | |
| --- | --- | --- | --- | --- | --- | --- |
|  | HR  (95% CI) | *P*-value | HR  (95% CI) | *P*-value | HR  (95% CI) | *P*-value |
| Class I | 1(Ref) |  | 1(Ref) |  | 1(Ref) |  |
| Classes II-III-IV | 6.2 (4.93~7.8) | <0.001 | 4.86 (3.85~6.13) | <0.001 | 2.06 (1.61~2.62) | <0.001 |

Model I: Unadjusted. Model II: Adjusted for age, sex, and race. Model III: Additionally adjusted for ASA physical status, anesthesia type, priority of surgery, and degree of preoperative anemia. The P for trend was calculated by treating the RCRI classes as an ordinal variable in the regression models. HR, hazard ratio; CI, confidence interval.


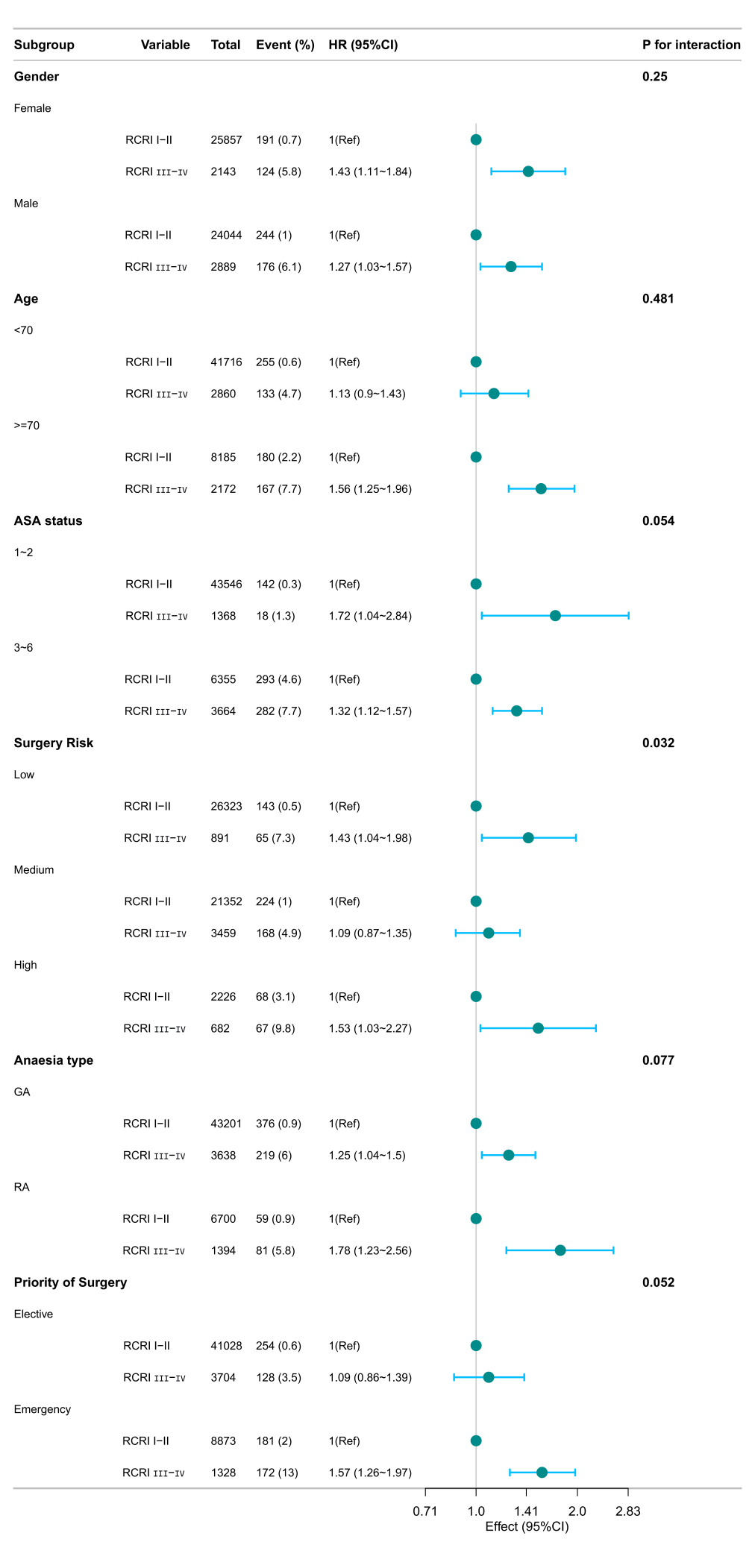


**Supplementary Figure S1. Forest plot for subgroup analysis of the association between RCRI (Classes I-II vs. III-IV) and 90-day mortality**

The plot displays hazard ratios (HRs, solid squares) and 95% confidence intervals (horizontal lines) for the association of RCRI Classes III-IV (vs. Classes I-II) with 90-day mortality across various patient subgroups. The size of the data markers corresponds to the precision of the estimate. Formal tests for interaction showed no significant effect modification by any subgroup variable (all p for interaction > 0.05), indicating a consistent association. GA, general anesthesia; RA, regional anesthesia.


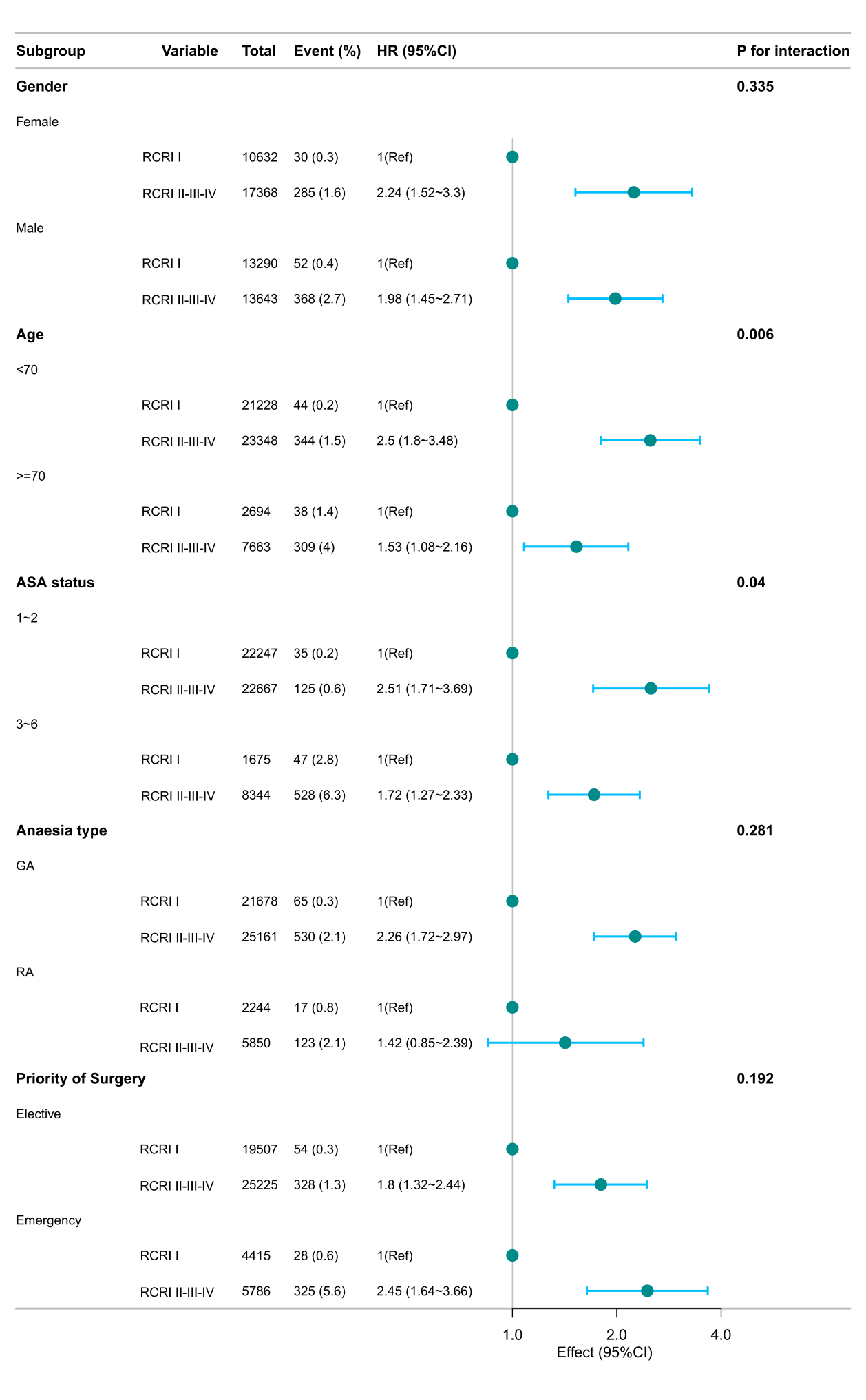


**Supplementary Figure S2. Forest plot for subgroup analysis of the association between RCRI (Class I vs. Classes II-III-IV) and 90-day mortality**

The plot displays hazard ratios (HRs, solid squares) and 95% confidence intervals (horizontal lines) for the association of RCRI Classes II-III-IV (vs. Class I) with 90-day mortality across various patient subgroups. The size of the data markers corresponds to the precision of the estimate. Formal tests for interaction showed no significant effect modification by any subgroup variable (all p for interaction > 0.05), indicating a consistent association. GA, general anesthesia; RA, regional anesthesia.

Note: Subgroup analysis by surgical risk category was not performed for this comparison because RCRI Class I patients were exclusively classified as low surgical risk (as shown in Table 1), leading to perfect collinearity.
